# Effective Treatment of Upper Urinary Tract Malignancies Using Laparoscopic Surgery in Three Nonagenarians in Poor General Condition: Are They Too Old for Surgery?

**DOI:** 10.1089/cren.2018.0059

**Published:** 2018-09-25

**Authors:** Takehiro Sejima, Shuichi Morizane, Katsuya Hikita, Masashi Honda, Atsushi Takenaka

**Affiliations:** ^1^ Department of Urology, Matsue City Hospital, Matsue, Japan.; ^2^ Division of Urology, Department of Surgery, Tottori University Faculty of Medicine, Yonago, Japan.

**Keywords:** laparoscopic surgery, renal malignancy, nonagenarian

## Abstract

***Backgrounds:*** Surgical procedures in the elderly are associated with higher perioperative morbidity and mortality rates than in younger patients. This is especially significant because elderly individuals are more likely to be operated on now than in the past because they represent the fastest growing subset of the population in advanced countries. Our cases are three nonagenarian patients with renal malignancy in poor general condition and were effectively treated by laparoscopic surgery.

***Case Presentation:*** Case 1 was a 91-year-old male patient with a right renal cell carcinoma of pT1b N0 M0. Case 2 was a 92-year-old male patient with a right renal pelvic tumor of pT3 N0 M0. Case 3 was a 90-year-old female patient with a left renal pelvic tumor of pT2 N0 M0. Case 1 had an Eastern Cooperative Oncology Group performance status of 1. The status of cases 2 and 3 was both rated as 2. All three cases had grade 3A chronic kidney disease. Cases 2 and 3 also had deep vein thrombosis in the lower extremities and dementia. In addition, case 2 had coronary occlusive disease. All cases were treated by laparoscopic surgery and effectively discharged from hospital without major physical complications.

***Conclusion:*** This report is the first English-language article that describes treating nonagenarian patients by laparoscopic urologic surgery. An increasing number of nonagenarian patients present with urologic malignancies, and surgeons are frequently faced with the question, “Are they too old for surgery?” Our report suggests that laparoscopic surgery for renal malignancy in nonagenarian patients is feasible.

## Introduction

Radical nephrectomy (RN) has been considered to be the most reliable treatment for renal tumors for decades. The traditional surgical procedure consists in removal of the entire kidney including Gerota's fascia, regional lymph nodes, and the adrenal gland. In the treatment of most clinical T1 renal masses, nephron-sparing surgery is now considered the appropriate treatment of choice, even in those with a normal contralateral kidney. Recently, laparoscopic RN has been widely recognized as the standard treatment because of its equivalence to open RN for oncologic control and survival rate.^1^ Elderly individuals are the fastest growing segment of the population. It is problematic for many clinicians to determine whether surgical intervention is appropriate in super-elderly patients with renal malignancy without metastasis because there is no effective treatment other than renal surgery. The number of reports of laparoscopic renal surgery in super-elderly patients is low. In this article, we report the effective treatment of three nonagenarian patients in poor general condition using laparoscopic RN and nephroureterectomy in Matsue City hospital.

## Case Presentations

### Case 1

**FIG. 1.**
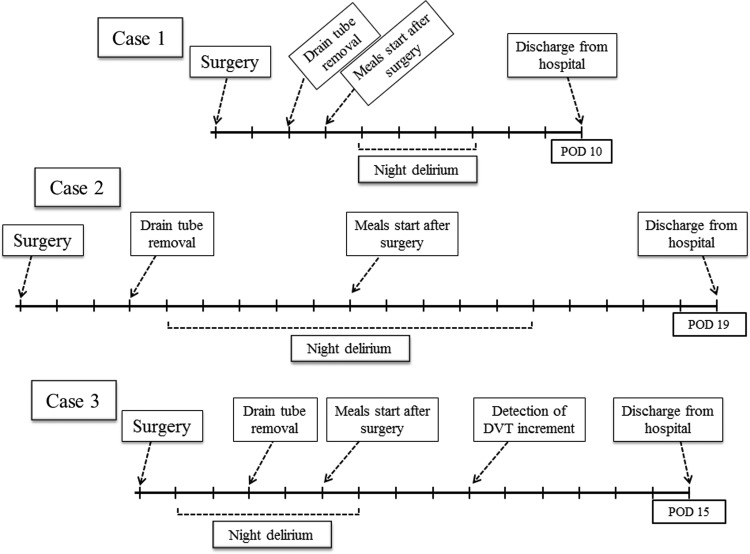
Hospitalized clinical course of three nonagenarian cases is shown. Postoperative night delirium occurred in all three cases and was effectively treated by administration of haloperidol and/or risperidone.

A 91-year-old male patient was referred to Matsue City hospital from a clinic for treating a right incidental renal tumor in November 2016. The case background is summarized in Table 1. Because the patient requested eradication of the disease, a laparoscopic RN procedure was performed in December 2016. Although surgical approach was conventional intraperitoneal approach, insufflation pressure was restricted <8 mm Hg considering advanced age. He experienced postoperative night delirium and was treated by administration of haloperidol. The patient was discharged on postoperative day (POD) 10 (Fig. 1). The pathologic diagnosis identified a multilocular clear cell renal cell carcinoma (RCC), Fuhrman grade 2, pT1b.

**Table 1.** Patient, Tumor, and Surgical Characteristics

*Characteristics*	*Case 1*	*Case 2*	*Case 3*
Age (years)	91	92	90
Gender	Male	Male	Female
Body mass index (kg/m^2^)	23.3	22.6	20
Eastern Cooperative Oncology Group performance status	1	2	2
Age-adjusted Charlson Comorbidity Index	7	10	9
American Society of Anesthesiologists score	Class II	Class III	Class II
Comorbidities	Chronic kidney disease (grade 3A)	Chronic kidney disease (grade 3A), DVT, dementia, coronary occlusive disease	Chronic kidney disease (grade 3A), DVT, dementia
Diseased side	Right	Right	Left
Clinical diagnosis	RCC	RCC	Renal pelvic tumor
Pathologic diagnosis	RCC	Urothelial carcinoma	Urothelial carcinoma
T (pathologic) NM	T1b N0 M0	T3 N0 M0	T3 N0 M0
Surgery time (minute) laparoscopic/total	147/201	192/232	150/260
Intraoperative blood loss (mL)	15	30	60
Surgical approach	Intraperitoneal	Intraperitoneal	Retropenitoneal
Postoperative complication	Night delirium	Night delirium	Night delirium, increment of DVT
Hospitalization period (day)	12	22	22

DVT = deep vein thrombosis; RCC = renal cell carcinoma.

### Case 2

**FIG. 2.**
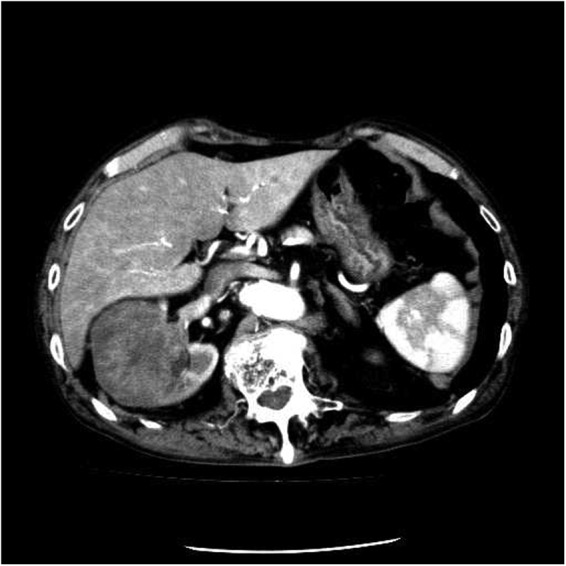
Enhanced CT scan of case 2 demonstrated a right renal tumor. The tumor was diagnosed as RCC by the radiologist who is the attending doctor and belongs to the Japan Radiological Society. RCC, renal cell carcinoma.

A 92-year-old male patient was referred to Matsue City hospital for treating a right renal tumor in February 2017. The case background is summarized in Table 1. The patient was in poor general condition with multiple comorbidities, which were considered to be critical obstacles for surgical treatment. Nevertheless, laparoscopic RN was performed in March 2017 because of the presence of progressive symptoms of a massive hematuria. Although surgical approach was conventional intraperitoneal approach, insufflation pressure was restricted <8 mm Hg considering advanced age. Postoperative continuous administration of heparin occurred for 7 days to prevent pulmonary infarction because of deep vein thrombosis (DVT) in lower extremities. Because the patient experienced postoperative night delirium, he was treated by administration of haloperidol and risperidone. The patient was discharged on POD 19 (Fig. 1). Although CT diagnosis was of right T3a RCC (Fig. 2), the pathologic diagnosis was invasive urothelial carcinoma, grade 3, pT3.

### Case 3

A 90-year-old female patient was referred to the Department of Urology from the Department of Neurology in Matsue City hospital for treating a left incidental renal pelvic tumor in July 2017. The case background is summarized in Table 1. The patient presented with a poor general condition and with multiple comorbidities, which were considered critical obstacles for surgical treatment. However, a laparoscopic radical nephroureterectomy was performed in August 2017 because of the urgent request for surgical treatment by the patient's daughter. Although surgical approach was a conventional retroperitoneal approach, insufflation pressure was restricted <8 mm Hg considering advanced age. Postoperative continuous administration of heparin was performed for 7 days to prevent pulmonary infarction because of DVT in the lower extremities. The patient experienced postoperative night delirium occurrence, and she was treated by administration of haloperidol. A postoperative ultrasonography of the lower extremities was employed to determine the increase of DVT. However, she complained of no symptoms caused by DVT in the lower extremities. She was discharged on POD 15 (Fig. 1). The pathologic diagnosis was invasive urothelial carcinoma, grade 2 > 3, pT3.

## Discussion

Many clinicians are ambivalent regarding the use of surgical treatments for super-elderly patients because it is uncertain whether critical adverse events may occur. It is extremely difficult to predict outcomes especially in nonagenarian patients. The sole quality analysis of surgery for nonagenarian patients was with respect to cholecystectomy. A retrospective study demonstrated the feasibility of cholecystectomy in 1007 cases with mortality and complication rates of 5.5% and 17.2%, respectively.^1^ Another retrospective study reported findings from the anesthesiologist's point of view regarding patients 100 years of age and older.^2^ To the best of our knowledge, our report is the first English article describing the treatment of nonagenarian patients by laparoscopic urologic surgery. Surgical type was determined to be laparoscopic, rather than open RN, in our cases because the superiority of a minimally invasive approach was evident. However, the length of hospital stay was extremely long in cases 2 and 3 compared with the average length of nonelderly patients treated by laparoscopic surgery because of the treatment of night delirium and the delayed recovery of activities of daily living. With regard to the length of hospital stay, the worldwide difference of social, economic, and health insurance system is influential. The average length of hospital stay of upper urinary tract malignancy patients treated by laparoscopic surgery is 1 week in our nation. The predominant influential factor that prolonged the length of hospital stay was the occurrence of night delirium in all three cases despite the absence of any major physical complications. Fortunately, all cases were effectively treated by administration of haloperidol and/or risperidone. It has been demonstrated that elderly patients tend to develop postoperative delirium because of life restrictions resulting from perioperative care and from the hospitalization itself. In the urologic surgery field, cognitive and functional statuses were demonstrated to be reliable predictors of postoperative delirium.^3^ Other predictors include age, history of hospitalization or delirium, intraoperative hypotensive episode, slow gait speed, and a rapid decline in renal function after surgery.^4^ Considering the postoperative events of our cases, it is suggested that a preoperative consult with a psychiatrist is necessary for the postsurgical management of super-elderly patients. Another problem was that tumor diagnosis was different between preoperative CT examination and postoperative pathology analysis in case 2. Because of the urgency of the patient's desire for relief from severe hematuria, lack of sufficient preoperative examinations may have contributed to the difference in diagnosis. Considering the Eastern Cooperative Oncology Group performance status, comorbidities, and symptoms from the tumor, case 2 was the most critical case. We were uncertain whether surgical treatment was appropriate. However, a prompt surgical plan was adopted because postponing surgery for additional preoperative examinations was not beneficial for the patient. In fact, it was thought that the patient was no longer able to tolerate the progressive hematuria at the time.

## Conclusion

Laparoscopic surgery for renal malignancy in nonagenarian patients is feasible. In addition to physical care, management of postoperative delirium should be performed throughout the treatment.

## Abbreviations Used

CT: computed tomography
DVT: deep vein thrombosis
POD: postoperative day
RCC: renal cell carcinoma
RN: radical nephrectomy
